# The Influence of κ-Carrageenan-R-Phycoerythrin Hydrogel on In Vitro Wound Healing and Biological Function

**DOI:** 10.3390/ijms241512358

**Published:** 2023-08-02

**Authors:** Selvakumari Ulagesan, Sathish Krishnan, Taek-Jeong Nam, Youn-Hee Choi

**Affiliations:** 1 Division of Fisheries Life Sciences, Pukyong National University, Nam-gu, Busan 48513, Republic of Korea; ula.selva@gmail.com; 2 Institute of Fisheries Sciences, Pukyong National University, Gijang-gun, Busan 46041, Republic of Korea; sathishmbt@gmail.com (S.K.); namtj@pknu.ac.kr (T.-J.N.)

**Keywords:** wound healing, κ-carrageenan, R-phycoerythrin, hydrogel, antioxidant activity, antimicrobial activity

## Abstract

Wound healing is widely recognized as a critical issue impacting the healthcare sector in numerous countries. The application of wound dressings multiple times in such instances can result in tissue damage, thereby increasing the complexity of wound healing. With the aim of tackling this necessity, in the present study, we have formulated a hydrogel using natural polysaccharide κ-carrageenan and phycobiliprotein R-phycoerythrin from *Pyropia yezoensis*. The formulated hydrogel κ-Carrageenan-R-Phycoerythrin (κ-CRG-R-PE) was analyzed for its antioxidant and antimicrobial activity. The wound healing potential of the κ-CRG-R-PE was evaluated in Hs27 cells by the wound scratch assay method. The hydrogel showed dose-dependent antioxidant activity and significant antimicrobial activity at 100 μg/mL concentration. κ-CRG-R-PE hydrogels promoted more rapid and complete wound closure than κ-Carrageenan (κ-CRG) hydrogel at 24 and 48 h. κ-CRG-R-PE hydrogels also filled the wound within 48 h of incubation, indicating that they positively affect fibroblast migration and wound healing.

## 1. Introduction

The skin, functioning as the human body’s primary defense system, safeguards against microbial infection and external environmental damage [1,2]. Skin defects are commonly caused by different internal and external factors, such as physical, chemical, thermal, mechanical, pressure, infection, disease, and other factors, leading to numerous injuries. Skin wounds are classified as acute or chronic depending on their origin and subsequent outcomes. Acute wounds progress through a sequence of molecular events, ultimately leading to the restoration of structural integrity, whereas chronic wounds remain unresolved and exhibit pathological processes, including ongoing inflammation, persistent infections, and necrosis [3]. Wound healing is a multifaceted physiological phenomenon encompassing various repair and reconstruction stages. Initially, it commences with the formation of blood clots, succeeded by the onset of inflammation. During this inflammatory phase, neutrophils and monocytes play a pivotal role in eliminating infections and releasing essential growth factors and biologically active substances necessary for tissue formation. Consequently, fibroblasts and endothelial cells undergo simultaneous proliferation within the wound space, initiating the process of angiogenesis. Ultimately, the differentiation of keratinocytes and follicular cells triggers the commencement of basement membrane reconstruction [4,5,6]. The requirement for an optimal wound care system cannot be overstated in promoting wound healing. This system must guarantee high humidity at the wound site, deliver non-toxic and non-allergenic care, absorb wound exudates, provide antimicrobial maintenance, and offer thermal insulation and soft mechanical support [7,8,9].

Bacterial infection poses a threat to both acute and chronic wounds, making them vulnerable to systemic infection, which can be life-threatening. Moreover, it hinders the healing process, causing prolonged inflammation [10]. In chronic wounds, persistent inflammatory responses lead to the buildup of reactive oxygen species (ROS). A balanced level of ROS is crucial for promoting angiogenesis at the wound site and regulating blood flow into the area. However, excessive ROS can act as secondary messengers, attracting immune cells to the wound site and inhibiting the transition from the inflammatory to the proliferative phase [11]. Consequently, an effective approach to accelerate wound healing could involve the application of substances that possess antimicrobial, antioxidant, and cell migratory properties. Antibacterial agents can prevent wound infections, while antioxidants can maintain ROS levels at non-toxic concentrations, thereby enhancing wound healing. Additionally, substances that stimulate the migration of dermal fibroblasts can expedite the re-epithelialization phase by promoting the movement of epithelial cells.

Hydrogel, a type of three-dimensional network formed by covalently or non-covalently linked polymers, possesses the capacity to absorb and retain a significant quantity of water while maintaining specific plasticity and elasticity [12]. With this aspect, hydrogels offer a moist environment for wound healing, possess the capacity to absorb wound exudate, and can be easily taken off without causing further injury [13,14,15]. Hydrogels present remarkable advantages for wound dressing due to the gentle conditions used in their processing and their ability to seamlessly incorporate various bioactive agents. Additionally, there are hydrogels that possess antibacterial and antifungal capabilities, offering potential advantages in the acceleration of wound healing [16]. Hydrogels, which can possess distinct physical and chemical properties, can be created using synthetic or natural polymers with a wide range of chemical compositions [17]. Among natural biopolymers, carrageenan has emerged as a highly suitable candidate for use in tissue engineering, wound healing, and drug delivery applications [18]. Graham et al. (2019) [19] describe carrageenan as a linear, sulfated, hydrophilic polysaccharide made up of disaccharide repeat units of galactose and (3,6)-anhydrogalactose. These units are connected by alternating α-(1,3)- and β-(1,4)-glycosidic links. Among the different types of carrageenan, κ-carrageenan (kappa) exhibits thermoreversible gelation when combined with potassium ions, while ι-carrageenan (iota) demonstrates the same behavior with calcium ions. The combination of carrageenan hydrogels with other polymers or molecules can effectively enhance the properties of a hydrogel under physiological conditions.

Phycobiliproteins (PBPs) exhibit a range of structurally and physiochemically conserved features. Moreover, they have been demonstrated to possess diverse biological activities, encompassing antioxidant, antibacterial, and antitumoral properties. These compelling attributes make PBPs an intriguing subject for various biotechnological applications in fields like biomedicine and food [20]. Cyanobacteria and micro- and macroalgae from the phylum Rhodophyta serve as the primary contributors to PBPs. Phycoerythrin, an abundant phycobiliprotein found in cyanobacteria, displays a natural red coloration. Phycoerythrin possesses numerous inherent pharmacological properties such as antioxidant activity, antimicrobial activity, anticancer activity, and anti-aging activity [21,22,23]. Additionally, this could be connected to its effectiveness in the wound healing process.

In the present study, κ-carrageenan (κ-CRG) hydrogel was developed with phycobiliprotein R-phycoerythrin (R-PE) extracted from *Pyropia yezoensis* and analyzed for wound healing activity.

## 2. Results

### 2.1. Synthesis and Physical Characterization of κ-Carrageenan-R-Phycoerythrin Hydrogels

**Figure 1:**
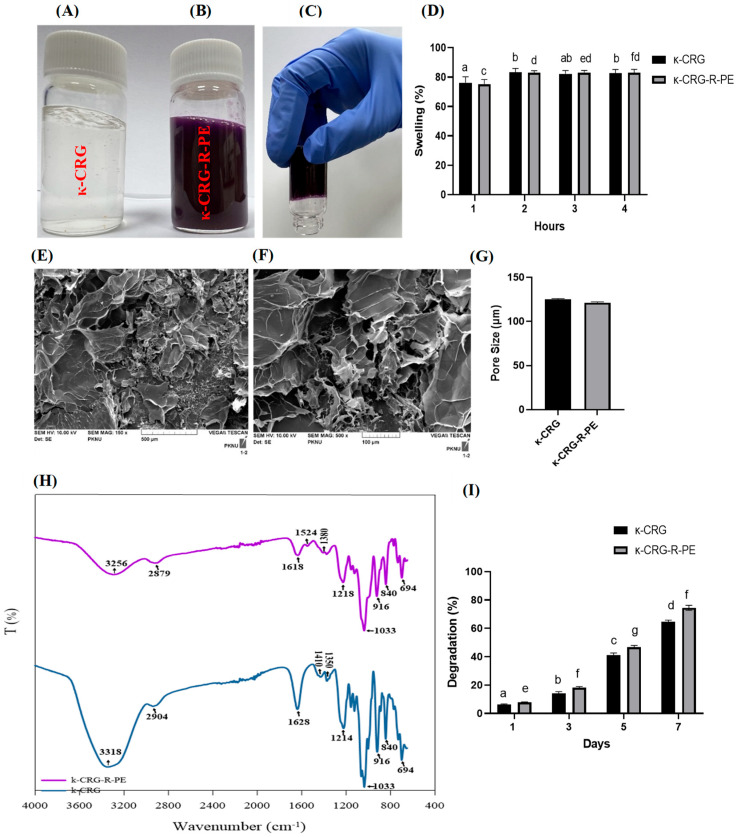
Synthesized hydrogel: (**A**) κ-carrageenan (κ-CRG) hydrogel, (**B**) κ-carrageenan-R-phycoerythrin (κ-CRG-R-PE) hydrogel, (**C**) Gelation property of the κ-carrageenan-R-phycoerythrin (κ-CRG-R-PE) hydrogel (**D**) Swelling analysis of hydrogels. Values are expressed as mean ± SD (*n* = 3). Different letters of the alphabet denote significant differences (*p* < 0.05), while the same letters of the alphabet indicate no significant difference (*p* > 0.05). (**E**) SEM image of κ-carrageenan (κ-CRG) hydrogel, (**F**) κ-carrageenan-R-phycoerythrin (κ-CRG-R-PE) hydrogel, (**G**) pore size of the hydrogels (**H**) FTIR spectra of κ-CRG hydrogel and κ-CRG-R-PE hydrogel (scale bar represents 100 μm), (**I**) Degradation analysis of hydrogels. Values are expressed as mean ± SD (*n* = 3). Different letters of the alphabet denote significant differences (*p* < 0.05), while the same letters of the alphabet indicate no significant difference (*p* > 0.05).

R-phycoerythrin was purified from the marine red algae *Pyropia yezoensis*. It was mixed with κ-carrageenan and synthesized as a hydrogel. The hydrogels were prepared using a solution comprising 1% κ-carrageenan and 1% R-PE dissolved in 5 mM KCl. After heating the κ-carrageenan solution to 80 °C for 30 min, it was allowed to cool down to 45 °C before the addition of the R-PE solution. The prepared hydrogels underwent incubation at 25 °C for 24 h, allowing the gel to cure and forming a uniformly structured gel [24]. The results are presented in Figure 1A–C. The gelation of κ-carrageenan involves a complex series of stages. Initially, when the polysaccharide solution is heated, it maintains a random coil conformation. Subsequently, a helical dimer is formed, which then aggregates with other helical dimers. Finally, a three-dimensional structure is established as the solution cools and cures at room temperature [25,26,27,28]. The swelling nature of the hydrogels κ-CRG and κ-CRG-R-PE was evaluated at 37 °C in PBS solution. The results obtained from the study regarding the percentage of swelling reveal that the examined hydrogels exhibit the capability to absorb substantial amounts of water. The hydrogels showed the following swelling percentages: κ-CRG 76.188 ± 6.1 at 1 h. and 82.76 ± 2.4 at 4 h, and κ-CRG-R-PE 75.27 ± 5.04 at 1 h. and 81.439 ± 2.3 at 4 h, respectively, as presented in Figure 1D. Hydrogel degradation was studied in the PBS for a duration of 7 days. By the end of this period, the degradation percentage of κ-CRG was observed to be 64.72 ± 5.1, whereas κ-CRG-R-PE demonstrated a degradation percentage of 73.91 ± 3.6, presented in Figure 1I. The Scanning Electron Microscope (SEM) analysis revealed a uniform interconnected porous system of the hydrogel (Figure 1E,F). The addition of R-PE did not result in significant variation in the SEM images. The introduction of κ-CRG in gel formation resulted in hydrogels with an average pore size of 125.174 ± 5.71 nm. The incorporation of R-PE did not show a noteworthy impact on pore size, as indicated by the average pore size of the κ-CRG-R-PE hydrogel, which was reported as 121.327 ± 7.57 nm (Figure 1G).

The freeze-dried hydrogels κ-CRG and κ-CRG–R-PE were analyzed for Fourier-Transform infrared spectroscopy (FTIR), as presented in Figure 1H. FTIR spectral analysis was performed to verify the appropriate functional groups presented in the pristine κ-carrageenan (κ-CRG) polymer and further the presence of encapsulated R-phycoerythrin (R-PE) into the κ-CRG. A sharp stretching band appeared at 1033 cm^−1^ and was assigned to the O=S=O stretching band. Moreover, the vibration bands at 916 cm^−1^, 840 cm^−1^, and 1218 cm^−1^ were assigned to the C-O, C-O-S, and sulfonyl group (SO3-) stretching bands of the κ-CRG units. After encapsulation of the R-PE units into the κ-CRG, the κ-CRG-R-PE gel samples showed that an additional stretching peak appeared at 1524 cm^−1^, indicating C-O and N-H stretching of the amide I and amide II groups. Furthermore, the additional vibration peaks appeared at 3256 cm^−1^ and 2879 cm^−1^, which represents the presence of carboxyl O-H and hydroxyl groups stretching the R-PE units. From the FTIR results, it could be confirmed that the R-PE units had been incorporated into the κ-CRG through electrostatic/H-bonding interactions.

### 2.2. Rheological Properties of Hydrogels

**Figure 2:**
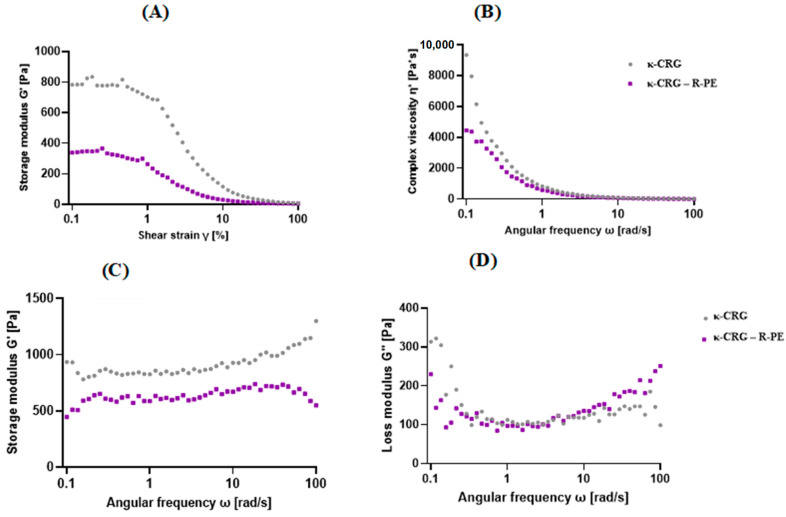
(**A**) Linear viscoelastic region (LVE) during an amplitude sweep test, (**B**) Complex viscosity (η*), (**C**) Storage modulus (G′), (**D**) Loss modulus (G″) during an angular frequency sweep test for hydrogels κ-CRG and κ-CRG-R-PE.

The rheological properties of the hydrogels were analyzed using a rheometer. Figure 2A shows the linear viscoelastic region (LVE) according to the increase in strain. The hydrogel’s reversible deformation was confined within the LVE (linear viscoelastic) range, but surpassing this limit resulted in the destruction of the sample structure [29]. The gel’s structural strength and elasticity are indicated by the storage modulus (G′), while its viscosity characteristics are denoted by the loss modulus (G″) (Figure 2B–D). According to the results of the amplitude sweep test, the hydrogels appeared as LVE at 0.3% strain, and this strain was applied to all subsequent experiments.

### 2.3. Antioxidant Activity

**Figure 3:**
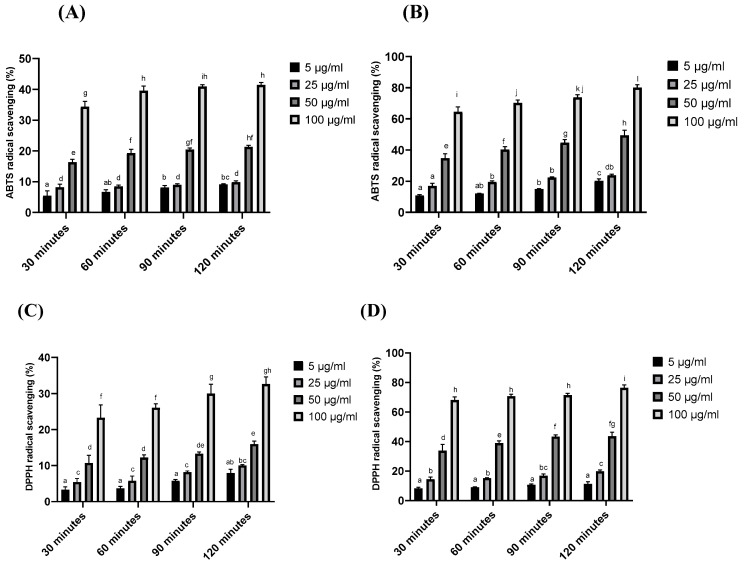
Antioxidant activity of κ-carrageenan (κ-CRG) hydrogel, κ-carrageenan-R-phycoerythrin (κ-CRG-R-PE) hydrogel: (**A**) ABTS radical scavenging activity of κ-CRG, (**B**) ABTS radical scavenging activity of κ-CRG-R-PE, (**C**) DPPH radical scavenging activity κ-CRG, (**D**) DPPH radical scavenging activity κ-CRG-R-PE. Values are expressed as mean ± SD (*n* = 3). Different letters of the alphabet denote significant differences (*p* < 0.05), while the same letters of the alphabet indicate no significant difference (*p* > 0.05).

The antioxidant activity of κ-CRG and κ-CRG–R-PE was determined using ABTS and DPPH assay. The results are presented in Figure 3A–D. Time-dependent radical scavenging activity was measured from 30 min to 120 min with different concentrations of hydrogels. The increasing concentration of the hydrogels showed increased free radical scavenging ability. In the ABTS assay, the scavenging ability of κ-CRG-R-PE hydrogel showed 80.09% at 100 μg/mL concentration in 120 min, while at the same concentration, the scavenging ability of κ-CRG hydrogel was only 41.4%. Similarly, in the DPPH assay, the scavenging percentage of κ-CRG-R-PE hydrogel was about 76.73% at 100 μg/mL concentration, while at the same concentration, the scavenging ability of the κ-CRG hydrogel was about 32.64%. Radical scavenging ability depends mainly on the R-phycoerythrin. From the results, it has been concluded that the combination of κ-CRG and R-PE effectively enhanced the antioxidant activity.

### 2.4. Antibacterial Assays (Well Diffusion Method)

**Figure 4:**
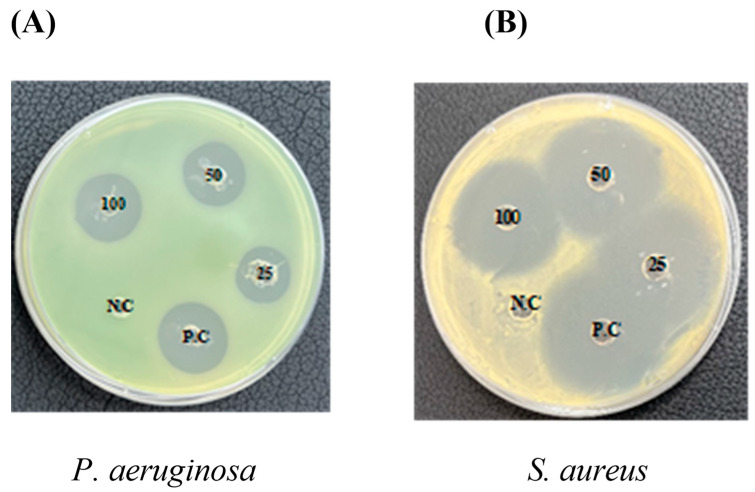
Antibacterial activity of κ-carrageenan (κ-CRG) hydrogel and κ-carrageenan-R-phycoerythrin (κ-CRG-R-PE) hydrogel: (**A**) Antibacterial activity against *P. aeruginosa*, (**B**) Antibacterial activity against *S. aureus*. Kanamycin was used as a positive control (50 μg/mL) and κ-carrageenan (κ-CRG) hydrogel (100 μg/mL) was used as a negative control.

Antibacterial activity κ-CRG and κ-CRG–R-PE was determined by the agar well diffusion method. The antibacterial activity was analyzed against gram-positive and gram-negative bacterial cultures such as *S. aureus* and *P. aeruginosa* (Figure 4A,B). The κ-CRG–R-PE hydrogel 100 μg/mL concentration showed a 15 mm zone of inhibition against *P. aeruginosa* and 25 mm against *S. aureus*, while κ-CRG hydrogel alone showed almost no antibacterial activity against the tested bacterial cultures. The results indicated that the coupling of κ-CRG and R-PE showed significant antibacterial activity (Table 1).

### 2.5. Cytotoxicity Studies

**Figure 5:**
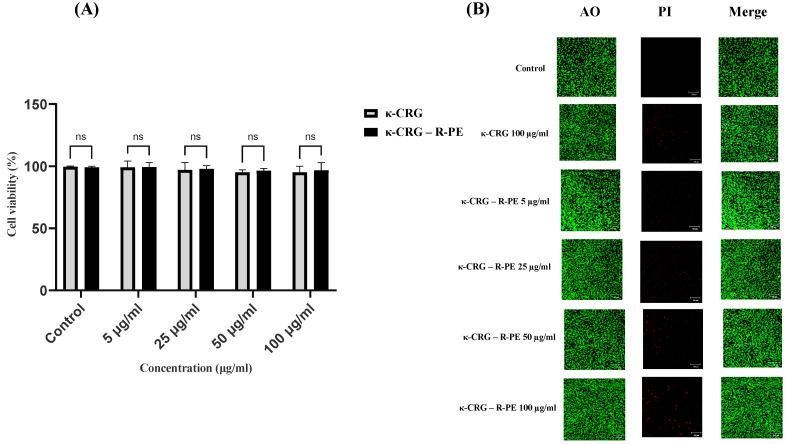
Cell viability assay and live–dead cell assay. (**A**) Cytotoxicity of κ-carrageenan (κ-CRG) hydrogel and κ-carrageenan-R-phycoerythrin (κ-CRG-R-PE) hydrogel analyzed in Hs27 cells. Values are expressed as mean ± SD *(n* = 3), ns (no significance). (**B**) Live–dead cell assay of Hs27 cells treated with different concentrations of hydrogels using propidium iodide and acridine orange staining. The scale bar represents 100 μm.

The cell viability assay indicated that the κ-CRG and κ-CRG-R-P hydrogels provide ECM-like support for cellular adhesion and growth. The maximum concentration of hydrogels (100 μg/mL) almost showed no toxicity against the cells. The κ-CRG hydrogel showed 94.90% and κ-CRG-R-PE hydrogel showed 96.6% cell viability (Figure 5A,B). From the literature review, it has been noted that the higher cell survival rate (>70%) exhibited no cytotoxicity [30]. The high biocompatibility of κ-CRG and the non-toxicity of R-PE resulted in good biosafety and certain application value. The live–dead assay results also supported the above results.

### 2.6. In Vitro Wound Healing

**Figure 6:**
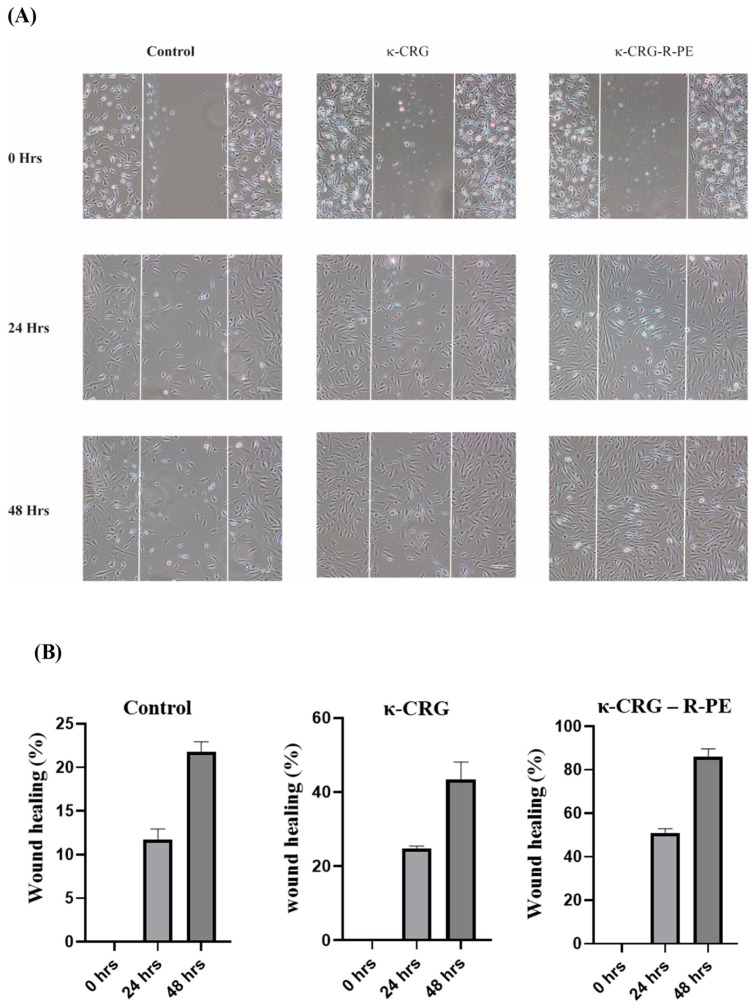
(**A**) Wound scratch assay showing gap closure at 0 h, 24 h, 48 h of control, κ-CRG hydrogel, κ-CRG-R-PE hydrogel, (**B**) Quantitative estimation of wound healing percentage in control, κ-CRG hydrogel, κ-CRG-R-PE hydrogel. The scale bar represents 100 μm. Values are expressed as mean ± SD (*n* = 3).

The wound healing activity of the synthesized hydrogels was analyzed by wound scratch assay and the results are presented in Figure 6A,B. A wound is created in the monolayer of the fibroblast, and, based on the cell migration and wound closure, it is considered as healed. The κ-CRG-R-PE hydrogel showed significant wound healing: a 100 μg/mL concentration showed 80% wound healing in 48 h when compared with the control and κ-CRG hydrogel.

## 3. Discussion

The present study elucidates the usefulness of a hydrogel prepared by the mixing of κ-carrageenan and R-phycoerythrin followed by an ionic cross-linking through KCl. Carrageenan exhibits gel formation in an aqueous solution as it transitions from a random coil to a helical conformation, subsequently leading to the aggregation of helices and the formation of a network [31]. Thus, the coil–helix transition is induced by cooling the solution in the presence of cations; potassium chloride promotes the gel formation of κ-CRG [31]. Moreover, an interconnected hydrogel allows cell growth and wound healing if it permits the continuous exchange of gases, nutrients, and metabolic waste products generally. To fulfill these requirements, the hydrogel should possess an interconnected network with controlled porosity and a pore size greater than 100 μm, as indicated by [32]. The SEM results and pore size of the hydrogels also confirmed the porous nature of the hydrogel with and without R-PE. The FTIR analysis showed that the characteristic absorption bands of the κ-CRG hydrogel and κ-CRG-R-PE hydrogel were observed in the carbohydrate fingerprint region, which is between 1300 and 800 cm. This region is known for its polysaccharide-specific bands, which have distinct positions and intensities [33]. Further, the incorporation of R-PE into κ-carrageenan resulted in the presence of new functional groups, such as amide I and amide II groups. Previous studies also confirmed the presence of the C-O and N-H stretching of amide I and amide II groups and the O=S=O stretching band from the R-PE [34,35]. The swelling percentage of the κ-CRG-R-PE showed a slight decrease, possibly attributed to enhanced ionic crosslinking within the network. The κ-CRG and κ-CRG-R-PE hydrogel, being endowed with a higher carrageenan content, resulted in an augmented electrostatic repulsion between sulfate groups. The effectiveness of κ-CRG in reducing syneresis results in a high water-holding capacity being attained [31]. Its high water-holding capacity proves advantageous in facilitating the transport of nutrients, products, or bioactive agents, including growth factors [36]. The κ-CRG-R-PE hydrogel demonstrated a slightly higher percentage of degradation compared to the κ-CRG hydrogel. For wound healing applications, hydrogels are purposely designed to degrade gradually, facilitating the controlled release of the bioactive substances or medications they contain. The degradation rate can be customized to suit the specific healing needs of the wound. This degradation process entails the hydrolysis or enzymatic cleavage of the polymer chains within the hydrogel. The biodegradation of biopolymers, like those used in hydrogels, involves a combination of enzymatic, microbial, and pH-driven actions, resulting in intricate biological, physical, and chemical processes [37].

The elastic behavior and structural strength of a material are reflected by the storage modulus, whereas its viscous response at a particular angular oscillation frequency [38] is represented by the loss modulus. In the present study, the rheological results of the hydrogels showed κ-CRG G′ > κ-CRG-R-PE G′, and G′ values show frequency-independent behavior for hydrogels. The addition of 1% R-phycoerythrin changed the stiffness of the gel. The κ-CRG-R-PE hydrogels showed a shear-thinning phenomenon in which complex viscosity decreased as frequency increased.

Further, the κ-CRG-R-PE hydrogel showed significantly higher antioxidant activity than the κ-CRG hydrogel alone. ABTS and DPPH assays indicated the higher scavenging ability in the κ-CRG-R-PE hydrogel in a dose-dependent manner. Previous studies also highlighted the antioxidant activity of R-PE [39,40]. Nevertheless, studies also mentioned the antioxidant activity of κ-CRG, which is limited when compared to the degraded, over-sulfated, acetylated, and phosphorylated derivatives of κ-CRG [41,42]. *Pseudomonas aeruginosa* and *Staphylococcus aureus* are the two most common causes of chronic wound infections [43,44,45,46]: the κ-CRG-R-PE hydrogel showed significant antimicrobial activity against *P. aeruginosa* and *S. aureus*. Additionally, previous research also emphasized that the R-PE exhibited strong inhibitory activity against *P. aeruginosa* (99.97%) and *S. aureus* (95.92%) with 0.1 mg/mL concentration [23,47]. The previous research highlighted that κ-CRG does not exhibit any antimicrobial activity against bacterial cultures, but the oxidized κ-carrageenan could damage the bacterial cell wall and cytoplasmic membrane and suppress the growth of both gram-positive and gram-negative bacteria [48]. The incorporation of phycoerythrin into the carrageenan hydrogel may enhance the stability of the pigment–protein complex and allow for a controlled release of the antibacterial compound over time. This sustained release of the antibacterial agent could lead to prolonged antimicrobial activity and the hydrogel matrix may provide a larger surface area for the phycoerythrin to interact with bacteria, potentially increasing its efficacy. Kanamycin is a widely studied and well-characterized antibiotic that exhibits potent antimicrobial activity against a broad range of bacterial strains. It is commonly used in clinical settings to treat various infections caused by susceptible bacteria [49]. In the present study, kanamycin was used as a positive control to compare the results.

The bioactivity of a material is generally related to its natural attributes and structural properties. Hydrophilic polymers with a rough surface and porous microstructure are thought to be more favorable for cell adhesion, migration, and proliferation [11,50]. The present study confirmed that the HS27 cell line showed no signs of toxicity at any of the concentrations tested, indicating the biocompatibility of the material for wound healing. Human dermal fibroblasts perform crucial functions in the skin, including collagen synthesis, extracellular matrix production, and wound healing facilitation. By studying their response to different substances, valuable insights can be gained regarding potential toxicity or adverse effects on these vital cellular processes. Moreover, fibroblasts are key contributors to the formation of newly synthesized tissue that fills wounds through the generation of necessary extracellular matrix. When tissue is injured, these cells initiate migration toward the affected area and aid in collagen deposition. Therefore, understanding the potential interactions between fibroblasts and wound dressings is of utmost importance, considering their vital role in the wound healing process. Ultimately, fibroblasts are essential for the proliferative phase, which is the final stage of wound healing [51]. They produce a fibrin matrix that can couple with κ-CRG-R-PE hydrogel to promote wound healing. This hydrogel might provide ECM-like support and helps fibroblasts migrate, which can accelerate wound healing [52,53]. κ-CRG-R-PE hydrogels promoted more rapid and complete wound closure than the κ-CRG hydrogel and the control at 24 and 48 h. κ-CRG-R-PE hydrogels also filled the wound within 48 h of incubation, indicating that they have a positive effect on fibroblast migration and wound healing. These findings are consistent with earlier reports, which have found similar results for phycocyanin-based hydrogel [54,55]. To the best of our knowledge, no previous studies have investigated the wound healing activity of R-PE.

## 4. Materials and Methods

### 4.1. Extraction and Purification of R-Phycoerythrin from Pyropia yezoensis

R-phycoerythrin (R-PE) from *Pyropia yezoensis* was extracted and purified as per the previous protocol [22]. Briefly, after sourcing the marine red algae *P. yezoensis* from Suhyup in Busan, South Korea, it underwent a process of freeze-drying and grinding, eventually becoming a powdered substance. To begin the extraction process, in a 500-mL flask, five grams of this powder were carefully suspended in 100 mL of ultrapure water. The suspension was subjected to sonication using the QSONICA sonicators (Model: USA Q500, Location: Newtown, CT, USA) utilizing 5 pulses, each lasting 5 s, and with a 5 s pause between each pulse. This sonication process was carried out for 1 h at 400 rpm under ice-cold conditions. Following sonication, the mixture was centrifuged at 45,000× *g* for 20 min at 4 °C. The resulting supernatant containing the soluble protein was succeeded by its precipitation through the addition of 80% ammonium sulfate.

Subsequently, the removal of ammonium sulfate from the precipitated pellet was effectively achieved by conducting dialysis with a 2 kDa dialysis membrane. The dialysis process was conducted against distilled water. Once the ammonium sulfate was completely removed, the soluble protein was collected and freeze-dried. Finally, the freeze-dried product was stored at 4 °C for future use.

### 4.2. Purification

The protein underwent several purification steps to obtain the final R-phycoerythrin alpha subunit. Initially, the separation of proteins was carried out using fast protein liquid chromatography with the AKTA Prime Plus system (GE Healthcare, Piscataway, NJ, USA). A HiPrep Sephacryl S-200 HR 16/60 column (GE Healthcare, Chicago, IL, USA) pre-equilibrated with 50 mM Tris-HCl at pH 7.2 facilitated the process. After injecting a 2 mL protein sample at a concentration of 1 mg/mL, a total of 80 fractions were collected. The peaks containing fractions were pooled together and subsequently analyzed using UV–Vis and fluorescence spectrometry techniques. Next, the purification of the protein was accomplished using reverse-phase high-performance liquid chromatography with a Sep-Pak plus C18 reversed-phase column (Waters, Milford, MA, USA). Reversed-phase buffer A with 0.1% (*vol*/*vol*) trifluoroacetic acid was employed to elute the bound protein with varying proportions of acetonitrile (20, 40, 60, or 80%). The protein solution was subjected to acetonitrile evaporation post-separation, and SDS-PAGE was employed to assess the protein content of each fraction. The phycoerythrin alpha subunit, with a molecular weight of 17.9 kDa, was identified as a single band. Finally, the resulting R-phycoerythrin alpha subunit was freeze-dried to preserve its stability.

### 4.3. Synthesis and Characterization of κ-Carrageenan-R-Phycoerythrin Hydrogels

A hydrogel composed of κ-carrageenan (κ-CRG) and R-phycoerythrin (R-PE) was fabricated using a 1:1 ratio (wt/wt) of κ-CRG and R-PE in 5 mM KCl. Initially, the κ-CRG solution was created and heated to 80 °C for 30 min in a water bath. After the complete dissolution of κ-CRG and the formation of a uniform solution, it was allowed to cool down, and R-PE was introduced. The resulting mixture was then uniformly dispersed through bath sonication and left to incubate at 25 °C (room temperature) for ionic crosslinking and gelation overnight [54]. These ionically crosslinked hydrogels were subsequently assessed for their physiochemical and biological properties.

Following the curing process, the gels were subjected to freeze-drying in a lyophilizer and subsequently sectioned into smaller pieces. These sections were then examined using scanning electron microscopy (SEM-VEGA, TESCAN 1-2, Brno–Kohoutovice, Czech Republic) to analyze the gel’s interconnected network. To enhance imaging, cross sections of the samples were affixed to carbon tape and coated with a layer of gold before capturing images at 10 kV with 500× magnification. The obtained SEM results were utilized to calculate the pore size of the hydrogels using ImageJ software 1.5.3. For FTIR analysis, the spectra were recorded using a JASCO-FT/IR-4100 spectrophotometer (Hachioji, Tokyo, Japan) over the range of 4000–600 cm^−1^. Prior to analysis, the lyophilized hydrogels were combined with potassium bromide (KBr) to form pellets.

#### 4.3.1. Swelling Analysis of Hydrogels

The swelling behavior of hydrogels was examined by immersing 5 mg of lyophilized hydrogels in 25 mL of PBS at 37 °C while maintaining their position. The weight of the hydrogels was measured at hourly intervals (ranging from 1 to 4 h) as they absorbed PBS and reached equilibrium, following the removal of excess PBS [56]. The percentage of swelling was determined using the following formula:

Swelling (g g − 1) = (Ws − Wd)/Wd × 100

where Ws represents the weight of the swollen hydrogel and Wd corresponds to the initial dry weight of the hydrogel.

#### 4.3.2. Biodegradation of Hydrogels

The investigation of hydrogel biodegradation involved immersing 25 mg of lyophilized hydrogels in 100 mL of Phosphate Buffer Saline (PBS). The hydrogels were subjected to a seven-day experiment in the PBS solution. Samples were collected at one, three, and seven days, and their respective weights were recorded. The degree of weight loss was calculated by subtracting the initial weight from the final weight [56].

The percentage of degradation was determined using the following formula:

Percentage of degradation = (Wi − Wf)/Wf × 100

where Wf represents the final weight of the hydrogel and Wi represents the initial weight of the hydrogel

#### 4.3.3. Rheological Properties of the Hydrogels

The hydrogels’ rheological properties were evaluated using an oscillatory test on a rheometer (MCR 92, Anton Paar Inc., Graz, Austria). All experiments utilized a parallel plate system with a 20 mm diameter and a 1 mm gap. Before measurement, the hydrogel samples were prepared and incubated at room temperature for 24 h. To determine the linear viscoelastic region (LVE), a strain sweep with a constant frequency (1 Hz) and log ramp strain (0.1–100%) was initially performed. The LVE is characterized by the storage modulus G′ and loss modulus G″ being independent of the applied strain for each hydrogel. Once the LVE was established for each hydrogel, a frequency sweep was conducted at a constant strain (0.1%). The frequency sweeps covered a log ramp frequency range of 0.1–10 Hz.

### 4.4. Antibacterial Activity

#### 4.4.1. Bacterial Strains

The antimicrobial efficacy of κ-CRG-R-PE hydrogel was assessed against both gram-negative (*Pseudomonas aeruginosa*) and gram-positive (*Staphylococcus aureus*) bacterial strains. Kanamycin served as the positive control in the experiments. The bacterial strains, *cm aureus* (KCTC 1621) and *Pseudomonas aeruginosa* (KCTC 1637), were procured from the Korean Collection for Type Cultures (KCTC).

#### 4.4.2. Antibacterial Assays (Well Diffusion Method)

The antibacterial activity was assessed using the agar well diffusion technique [57]. To begin, Mueller–Hinton agar medium (MHA) was prepared at a pH of 7.4 and sterilized through autoclaving at 121 °C and 15 psi for 15 min. Sterilized Petri dishes were filled with 20 mL aliquots of the prepared MHA and left to solidify at room temperature. Using a sterile cotton swab, each test organism from a 24 h inoculated broth was evenly spread on the MHA plates. After a short waiting period to ensure complete absorption of the inoculum, a 5 mm diameter well was created at the center of each plate using a sterilized cork borer. For the experimental groups, different concentrations (25, 50, and 100 µg/mL) of κ-CRG-R-PE were added to the corresponding wells in the MH agar plates. A positive control of kanamycin at a concentration of 50 µg/mL and a negative control of κ-CRG (100 µg/mL) were included. Following the addition of κ-CRG-R-PE or kanamycin, the plates were incubated at 37 °C for 24 h. The presence of antimicrobial activity was indicated by the appearance of a clear inhibition zone around the well. The zone of inhibition was measured using an antibiotic zone measuring scale, and all results were recorded in triplicate.

### 4.5. Antioxidant Activity

#### 4.5.1. DPPH Assay

The radical scavenging activity of κ-CRG and κ-CRG–R-PE was assessed using 1,1-diphenyl-2-picryl hydroxyl (DPPH) as the scavenger agent [58]. In brief, a 0.1 mM ethanol solution of DPPH was prepared. Subsequently, 50 μL of this DPPH solution was combined with 150 μL of a solution containing 5, 25, 50, and 100 μg/mL of κ-CRG and κ-CRG–R-PE, respectively. The mixture was then incubated at room temperature in the dark for a duration of 30–120 min. During the incubation period, the absorbance of the mixture was measured at 517 nm at different time intervals using a UV–Vis spectrophotometer (Shimadzu). As a reference standard, ascorbic acid was employed. The percentage of DPPH scavenging was determined using the following equation:

DPPH scavenging effect (%) or percent inhibition = (A0 − A1)/A0 × 100

where A0 represents the absorbance of the control reaction and A1 represents the absorbance of the DPPH assay solution in the presence of the test or standard sample. The experimental results were obtained in triplicate to ensure accuracy.

#### 4.5.2. ABTS Assay

The experimental procedure for the ABTS assay followed the methodology outlined in reference [59]. A stock solution of 7 mM ABTS and 2.4 mM potassium persulfate was prepared separately and mixed in equal volumes. This mixture was allowed to react in the dark for 12–16 h. After the incubation, the ABTS solution was diluted with methanol to achieve an absorbance of 0.700 ± 0.03 at 734 nm. To evaluate the ABTS radical scavenging activity, κ-CRG and κ-CRG–R-PE were added at different concentrations (5, 25, 50, and 100 μg/mL) to 2 mL of the ABTS solution. The absorbance of each mixture was measured at 734 nm during incubation at room temperature and at various time intervals (30–120 min). Ascorbic acid served as the reference standard. The calculation for ABTS radical scavenging activity (%) was performed using the formula:

ABTS radical scavenging activity (%) = (A0 − A1)/A0 × 100

where A0 represents the absorbance of the control reaction and A1 is the absorbance of the ABTS assay solution in the presence of the test or standard sample. All experimental measurements were conducted in triplicate.

### 4.6. Cell Culture

The Hs27 human skin fibroblast cell line (CRL-1634-ATCC) was obtained from the American Type Culture Collection (ATCC, Manassas, VA, USA). The cells were cultured in complete Dulbecco’s modified Eagle’s medium (DMEM) supplemented with 10% fetal bovine serum (FBS), 100 U/mL penicillin, and 100 mg/mL streptomycin. Culturing was performed in a humidified 5% CO_2_ incubator at 37 °C until the cells reached 70–80% confluence in a 100 mm diameter plate. The cells were used between passages 5 and 15, with the medium being refreshed every 2 days.

#### 4.6.1. MTS Assay

Cell viability was assessed utilizing the CellTiter 96 aqueous non-radioactive cell proliferation assay (Promega). This assay is based on the conversion of 3-(4,5-dimethylthiazol-2-yl)-5-(3-carboxymethoxyphenyl)-2-(4-sulfonyl)-2H-tetrazolium (MTS) into a formazan product. Hs27 cells at a density of 1 × 10^4^ cells/well were plated in 96-well plates with 100 µL of DMEM supplemented with 10% FBS and incubated at 37 °C for 24 h to facilitate surface attachment. The medium was then replaced with varying concentrations of freeze-dried hydrogels κ-CRG and κ-CRG–R-PE suspended in a serum-free medium (at least three wells per concentration). After 24 h of incubation at 37 °C, the cells were exposed to 10 µL of MTS solution for 30 min at 37 °C, and the absorbance was measured at 490 nm using a microplate reader (Benchmark microplate reader; Bio-Rad Laboratories, Hercules, CA, USA) [60]. Cell viability was determined by comparing the absorbance of treated cells to that of untreated cells. Each experiment was performed in triplicate.

#### 4.6.2. Live Dead Assay

##### Acridine Orange (AO) and PI Staining

Hs27cells were seeded in six-well plates, treated with κ-CRG–R-PE (5, 25, 50,100 μg/mL), washed twice with ice-cold PBS, stained with AO/PI (1 mg/mL each), and incubated at RT for 20 min in darkness. After washing off the stain with ice-cold PBS, cells were examined under a fluorescence microscope.

#### 4.6.3. In Vitro Wound Healing Scratch Assay

The wound scratch assay method was employed to examine in vitro wound healing. Human skin fibroblast Hs27 cells were seeded at a concentration of 1 × 10^5^ cells/well in 24-well plates containing 500 µL of DMEM supplemented with 10% FBS. The cells were then incubated at 37 °C for 24 h to facilitate surface attachment. Subsequently, the medium was replaced with serum-free media, and the cells were further incubated for 12 h at 37 °C to induce starvation conditions, thus reducing cell proliferation. Following the starvation period, a scratch was created on the cell monolayer using a 200 μL pipette tip to simulate a scratch wound. The scratched cells were then washed twice with 1XPBS to eliminate the population of cells that were displaced during the scratch. The scratched cells were treated with hydrogels κ-CRG and κ-CRG–R-PE, which were previously suspended in a serum-free medium at a concentration of 50 μg/mL. The cell layer was periodically imaged at 0, 24, and 48 h using phase-contrast microscopy. To quantify the wound healing progress, the percentage of wound healing was calculated by measuring the area of the wound covered using ImageJ software.

### 4.7. Statistical Analysis

The statistical analysis was performed using Origin 8.5 software (OriginLab Corporation, Northampton, MA, USA). The outcome is presented as mean ± standard deviation. To assess statistical significance, one-way analysis of variance (ANOVA) with post hoc multiple comparisons was applied, considering ** *p* < 0.01 or * *p* < 0.05 as statistically significant.

## 5. Conclusions

In the current study, the R-phycoerythrin-based κ-CRG hydrogel showed significant antioxidant and antimicrobial activity. The hydrogel’s macroporous structure was well-distributed and interconnected, making it highly suitable for advanced wound care due to its superior biocompatibility. It has been discovered through these findings that κ-CRG-R-PE can potentially influence wound healing. Continued research is essential to investigate in vivo wound healing.

## Figures and Tables

**Table 1:** Antibacterial activity of κ-carrageenan (κ-CRG) hydrogel and κ-carrageenan-R-phycoerythrin (κ-CRG-R-PE) hydrogel.

Micro-Organisms	Zone of Inhibition in mm				
Concentration of κ-CRG—R-PE	25 µg/mL	50 µg/mL	100 µg/mL	P.C	N.C (κ-CRG)
*P. aeruginosa*	8 ± 0.5 **	12 ± 0.72 **	15 ± 0.7 *	22 ± 0.5	-
*S. aureus*	20 ± 1 **	22 ± 1.2	25 ± 0.75 *	27 ± 1	-

Data are expressed as the means of triplicate measurements ± SD. Kanamycin was used as a positive control (50 μg/mL) and κ-carrageenan (κ-CRG) hydrogel (100 μg/mL) was used as a negative control. ** indicates significant differences (*p* < 0.005), while * indicates significant difference (*p* < 0.05).

## Data Availability

The data presented in this study are available from the authors.
